# Interactions between immune challenges and cancer cells proliferation: timing does matter!

**DOI:** 10.1093/emph/eow025

**Published:** 2016-08-18

**Authors:** Camille Jacqueline, Youssef Bourfia, Hassan Hbid, Gabriele Sorci, Frédéric Thomas, Benjamin Roche

**Affiliations:** 1 CREEC, 911 Avenue Agropolis, BP 64501, Montpellier, Cedex 5 34394, France; 2 MIVEGEC, UMR IRD/CNRS/UM 5290, 911 Avenue Agropolis, BP 64501, Montpellier, Cedex 5 34394, France; 3 Laboratoire Jacques-Louis Lions (LJLL), UMR 7598 Université Pierre et Marie Curie (UPMC), Paris 6, Boîte courrier 187, ;Paris, Cedex 05 75252, France; 4 Université Cadi Ayyad Laboratoire de Mathématiques et Dynamique de Populations, Cadi Ayyad University, Marrakech, Morocco; 5 International Center for Mathematical and Computational Modeling of Complex Systems (UMI IRD/UPMC UMMISCO), 32 Avenue Henri Varagnat, Bondy Cedex 93143, France; 6 BiogéoSciences, CNRS UMR 6282, Université de Bourgogne, 6 Boulevard Gabriel, Dijon 21000, France

**Keywords:** infectious diseases, immunosenescence, immunosuppression, cancer

## Abstract

The immune system is a key component of malignant cell control and it is also involved in the elimination of pathogens that threaten the host. Despite our body is permanently exposed to a myriad of pathogens, the interference of such infections with the immune responses against cancer has been poorly investigated. Through a mathematical model, we show that the frequency, the duration and the action (positive or negative) of immune challenges may significantly impact tumor proliferation. First, we observe that a long immunosuppressive challenge increases accumulation of cancerous cells only if it occurs 14 years after the beginning of immunosenescence. However, short immune challenges result in an even greater accumulation of cancerous cells for the same total duration of immunosuppression. Finally, we show that short challenges of immune activation could lead to a slightly decrease in cancerous cell accumulation compared to a long one. Our results predict that frequent and acute immune challenges could have a different and in some extent higher impact on cancer risk than persistent ones even they have been much less studied in cancer epidemiology. These results are discussed regarding the existing empirical evidences and we suggest potential novel indirect role of infectious diseases on cancer incidence which should be investigated to improve prevention strategies against cancer.

## Teaser

Using first a theoretical framework, we show that repeated short immune challenges could impact the accumulation of cancerous cells through continuous perturbation of immune system efficiency. We discuss for a new indirect role for infectious disease in cancer progression.

## INTRODUCTION

While cancer remains one of the main causes of death in Western countries [1], its burden is increasing in low- and middle-income countries [2]. Today, the most common approach for removing cancerous cells is to treat affected individuals through surgery, cytotoxic drugs and/or radiotherapy [3]. Nevertheless, immunotherapy [4], aiming to stimulate the immune system to improve the control of cancerous cell proliferation [5], holds promise to be an alternative of current classic therapies.

In fact, the immune system has three major roles in cancer suppression [6,7]. First, it can eliminate oncogenic pathogens (i.e. infectious organisms recognized to have a contribution to carcinogenesis [8]) and therefore protect the host from developing the associated tumor. Second, it can prevent pro-tumoral inflammatory environment by resolving inflammation right after pathogen elimination [9]. Finally, immunosurveillance bring into play many cells both from innate and adaptive immunity, especially T cells [7], that eliminate tumor cells and produce signaling molecules (cytokines) at both the tumor and peripheral sites [10].

However, it is now well recognized that immune system fails to avoid cancer proliferation and can have a paradoxical role which has been explained by the immunoediting hypothesis [11]. At some point, immune clearance is switched to escape mechanisms, such as recruitement of immunosuppressive cells, allowing an increase in cancerous cell accumulation in late cancer stage [12]. Immune cells can also promote angiogenesis, produce growth factors and increase chronic inflammation in the tumor microenvironment which are considered as hallmarks of cancer and could result in activation of premalignant lesions [11, 13].

Humans are probably exposed to a high number of immune challenges (through contact, ingestion and inhalation) [14] which could impact the roles of the immune system in carcinogenesis. Especially, our immune system is implicated in control/elimination of intra- and extra-cellular infectious agents through a complex network of interdependent immune pathways that also involves adaptive immunity against cancerous cells control [15]. However, the role of immune challenges following infections, which could divert adaptive immunity against cancerous cells, has been poorly investigated.

First, infections could have a detrimental role for the host by reducing immune responses against cancerous cells. In fact, they can induce immunosuppression, here defined as a decrease in efficiency of innate and adaptive actors (due to depletion of dividing cells for instance). HIV infection is one of the most well-known examples of an immunosuppressive virus as it depletes CD4^+ ^T cells [16]. These CD4 helper T cells produce high level of IFN-γ, as well as chemokines, that enhance the priming and expansion of CD8 cytotoxic cells which eliminate cancerous cells [17]. Helminths species are also able to impair immune efficiency through their immunoregulatory roles [18]. In fact, helminths are known to inhibit T cell proliferation and to promote expansion of Treg cells which are able to impede effective immunity against cancer by secreting TGF-β [17].

Second, infections are known to induce adaptive immune responses that could boost the elimination of cancerous cells. Early after an infection, the quantity of humoral and cellular effectors increases during acute inflammation and could cross react with tumoral antigens [19, 20]. In addition, the well-known trade-off between the Th1/Th17 and Th2 immune pathways suggests that Th1 or Th2 cytokines are able to downregulate each other and the associated humoral and cellular effectors [21]. However, Th1 activation is associated with protection against some cancers [22, 23]. In fact, it results in recruitment of natural killer (NK) cells and type I macrophages to tumor sites, which can act in concert toward tumor control [24]. Thus, all the infections that activate Th1 could reduce cancerous cell accumulation.

The timing of these immune challenges may also be crucial since our immune system is not permanently fully efficient. Indeed, immunosenescence is a process that reflects a gradual decrease of immune system activity with age mainly through a decreased capacity of immunosurveillance [25]. The beginning of immunosenescence is assumed to be associated with the beginning of thymopoiesis decline. Indeed, the thymus play a crucial role in the development of T cells but also in maintaining immune efficiency [26]. Maximal activity is reached at puberty (from 10 to 19 years old according to the World Health Organization) and decrease progressively in adults [27]. The elderly (>65 years old; WHO) usually have the following: (i) a depleted population of naïve T cells (the set of T lymphocytes that can respond to novel antigens) [28, 29], (ii) a shrinking repertoire of T cell clones [28, 30, 31], (iii) an increased number of naturally occurring regulatory T cells that down-regulate T cell responses [32, 33], (iv) a low grade, pro-inflammatory status [29] and (v) increased numbers of myeloid-derived suppressor cells, which are associated with impaired T-cell functioning and produce high amounts of reactive oxygen species [34]. All these immune-associated changes can potentially promote tumor proliferation [31].

While the role of immunosenescence on cancer development has already been suggested [28], the combination of this long-term irreversible process with sporadic, transient immune challenges has rarely been considered. In this article, we explore theoretically the combined role of immunosenescence with both persistent and repeated acute immune challenges on proliferation of cancerous cells. To this purpose, we consider that challenges could reduce or boost immune responses against cancerous cells. We also discuss the potential consequences of our findings in terms of cancer prevention.

## MATERIALS AND METHODS

We explored the combined influence of immunosenescence with sporadic and partial alteration of immune system functioning on the accumulation of cancerous cells through the following theoretical framework:

$$\frac{dH}{dt}=\beta_{1}H\left[1-\frac{N}{K}\right]-\mu_{1}H$$

$$\frac{dP}{dt}=\beta_{1}P\left[1-\frac{N}{K}\right]+\mu_{1}H-\left(\mu_{2}+\omega \left(t\right)\right)P$$

$$\frac{dC}{dt}=\beta_{2}C\left[1-\frac{N}{K}\right]+\mu_{2}P-\left(\mu_{3}+\omega \left(t\right)\right)C$$

$$\frac{dI}{dt}=\beta_{2}I\left[1-\frac{N}{K}\right]+\mu_{3}C$$

where *H* represents healthy cells, *P* precancerous cells, *C* cancerous cells and *I* cancerous cells that are invisible to the immune system. In a sequential manner, healthy cells become precancerous at rate *μ*_1_ (cells which for a precancer and respect the following criteria: (i) they increase the risk of cancer; (ii) cancerous cells arise from precancerous cells and (iii) precancerous cells are different from cancerous cells and normal cells but share some of their molecular and phenotypic properties [35]). Then, precancerous cells become cancerous at rate *μ*_2_, and finally invisible at rate *μ*_3_. We consider that invisible cancerous cells have acquired the capacity to avoid destruction by immune system whatever the mechanism implied (e.g. loss of MHC molecules and secretion of cytokines). Healthy and precancerous cells replicate at rate *β*_1_ while cancerous and invisible cells replicate at rate *β*_2_ (greater than *β*_1_) with a maximal total number of cells *K* (i.e. carrying capacity) in order to induce competition between different kinds of cells. We assumed that cancerous cells (C and I) are autonomous and do not depend of precancerous cells to survive. Such assumption could have an impact only if precancerous cells disappeared from the population, which is unlikely to occur with our parameters chosen in accordance with the available literature (Table 1).

**Table 1.** Parameters values used to model dynamics of cells and immune system efficiency. Parameters are defined in the text

Parameter	Definition	Value	Additional information	Reference
*β*_1_	Replication rate of healthy cells and precancerous cells	[0.45–1.2] cell per day	20–53H (example for gastric tissues)	[36]
		mean=0.82 cell per day		
*β*_2_	Replication rate of nonhealthy cells	[0.46–1.8] cell per day	13–52H (example for gastric tissues)	[36]
		mean=1.13 cell per day		
*K*	Carrying capacity of the tissue	10^13^	Assuming the total number of cells in human body is 3.72×10^13^	[37]
*μ*_1_	Mutation rate from healthy to pre-cancerous cell	2.99×10^−6^ per year	Based on Human mutation rate (10^−8^ generation) and 299 cell generation per year	[38]
*μ*_2_	Mutation rate from pre-cancerous cell to cancerous cell	2.99×10^−6^ per year	Idem	[38]
*μ*_3_	Mutation rate from cancerous cell to invisible cell	4.12×10^−6^ per year	Based on human mutation rate (10^−8^ generation) and 412 cell generation per year	[38]
*b*_0_	Beginning of immunosenescence	20 years	The thymopoiesis starts to decline in healthy adults after 20 years	[27]
*a*_1_	Immune efficiency before immunosenescence	0.7	Fixed	
*a*_2_	Rate at the immune system’s efficiency decreases	0.003 per year	Fixed to have a 70% reduction over 50 years of immunosenescence, as documented for the decrease of B cell stimulation in ederly individuals	[39]
*a*_3_	Amplitude of immune alteration	±0.7	Fixed	

**Figure 1.**
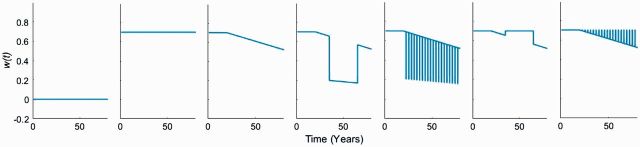
Examples of different immune system activity across ages (0–80 years). First column (*a*_1 _= 0), second column (*a*_1 _= 0.7, *a*_2 _= 0), third column (*a*_1 _= 0.7, *b*_0 _= 20 years, *a*_2 _= 3×10 ^−^^3^), fourth column (*a*_1 _= 0.7, *b*_0 _= 20 years, *a*_2 _= 3×10 ^−^^3^, *a*_3 _= 0.7; total duration = 30 years), fifth column (*a*_1 _= 0.7, *b*_0 _= 20 years, *a*_2 _= 3×10 ^−^^3^, *a*_3 _= 0.7; total duration = 4 years, 20 episodes of 70 days), sixth column (*a*_1 _= 0.7, *b*_0 _= 20 years, *a*_2 _= 3×10 ^−^^3^, *a*_3_ = −0.7; total duration = 30 years), seventh column (*a*_1 _= 0.7, *b*_0 _= 20 years, *a*_2 _= 3×10 ^−^^3^, *a*_3_ = −0.7; total duration = 4 years, 20 episodes of 70 days)

Each precancerous and cancerous cells can be eliminated from the organism through the function *ω(t)*. This function, temporally forced, aims to mimic the efficiency of the immune system during the lifetime of the organism considered. Five main parameters describe this function:

$$w\left(t\right)=a_{1}ift<b_{0}$$

$$w\left(t\right)=a_{1}-a_{2}tift>b_{0}$$

$$w\left(t\right)={(a}_{1}-a_{2}t)a_{3}ifc_{n}>t>d_{n}$$

$$withw(t)<a_{1}$$

where *a*_1_ represents the immune system efficiency before the beginning of immunosenescence (occurring at time *b*_0_). When immunosenescence starts, we assume that the immune system’s efficiency decreases linearly with time through a coefficient *a*_2_. As the number of immune challenges encounter in one life is particularly hard to determine, we choose a restrictive number of 30 challenges from ages 20 to 80 years. During an immune challenge *n* (starting at time *c_n_* and ending at time *d_n_*, thus for a duration *d_n_*−*c_n_*), the immune system efficiency is multiplied by a proportion *a*_3_ that characterizes the amplitude and the direction of this immune system alteration (with −1 < *a*_3 _< 1; allowing a gradual efficiency from immunosuppression when 0 < *a*_3 _< 1 with a positive impact on cancerous cells proliferation to a negative impact through immune system activation when −1 < *a*_3 _< 0). We assume that these immune challenges occur evenly between the beginning of immunosenescence and the end of life. In other words, the duration between each challenge will be identical. This flexible function allows us to study different scenarios of temporary and partial alteration of the immune efficiency which few of them are illustrated in Fig. 1. While our theoretical framework can address a gradient in the duration of immune challenges, we consider that an acute immune challenge lasts for <6 months whereas persistent ones alter immune system for a longer period of time.

We explore the respective contribution of the duration and the frequency of immune challenges on the number of cancerous cells at the age of 80 years (assumed to be the end of individual’s life), used as an estimation of cancer risk. We start all our simulations by considering that individuals have only healthy cells (S = K, *P* = 0; C = 0; I = 0). Finally, we test for the sensitivity of these impacts through a Latin Hypercube Sampling [40] with 100 iterations that allows exploring the robustness of our conclusion by adding uncertainties around parameters values.

## RESULTS

### Influence of timing and duration of a single immunosuppressive challenge

**Figure 2.**
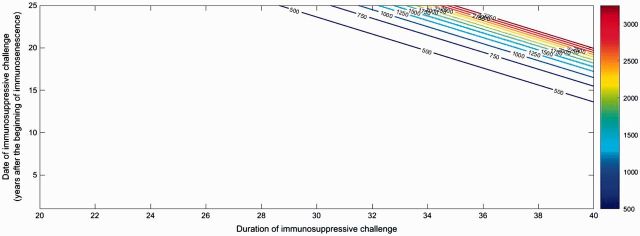
Contour plot of the number of cancerous cells at 80 (ranging from dark blue for accumulation of < 500 cancer cells to dark red for situations with >3000 cells) according to the date of an immunosuppressive infection after the beginning of immunosenescence and its duration. The maximal number of cancerous cells accumulates for 20 challenges with a total duration of 40 years (i.e. 3311 cells). Parameters are detailed in Table 1

We first aimed to quantify the influence of the duration and timing of a single immunosuppressive challenge on cancerous cell accumulation at the end of individual life. Figure 2 shows that a long episode of immunosuppression leads to large accumulations of nonhealthy cells by avoiding their elimination by the immune system.

Our theoretical framework also shows that the timing of the challenge through the lifespan is worth of consideration. In fact, Figure 2 highlights that a persistent immunosuppressive challenge occuring before immunosenescence will not significantly impact cancerous cell accumulation even if it persists during 40 years. To have a significant increase of nonhealthy cells at the age of 80 years, the challenge must occur at least 14 years after the beginning of immunosenescence. Since the immune system is weaker at this time than before immunosenescence, numerous cancerous invisible cells may have emerged during the immunosuppressive challenge, yielding a continuous proliferation of these cells, even when the individual recovers. In addition, even if the immunosuppressive challenge occurs 25 years after the beginning of immunosenescence, it will have an impact on cancerous cell accumulation only if it persists 29 years.

### Combined effect of duration and the number of immunosuppressive challenges

We then explored the combined influence of the duration and number of immunosuppressive challenges on cancerous cell accumulation. As previously said, we assumed that challenges are evenly distributed after the beginning of immunosenescence.

**Figure 3.**
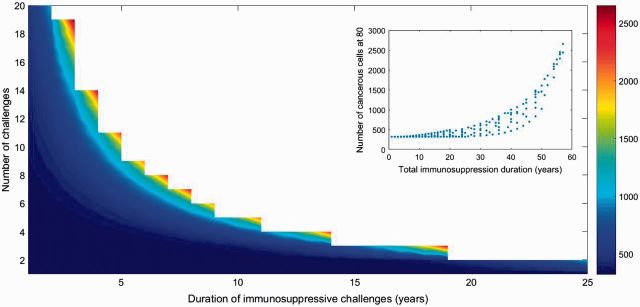
Influence of the number of immunosuppressive infections and their duration on the accumulation of cancerous cells (range from dark blue for accumulation of < 500 cancer cells to dark red of > 2500 cells). White area represents parameters sets where total immunosuppression period is > 60 years. The maximal number of cancerous cells accumulated at 80 is of 2653 cells. (Inplot) Relationship between total immunosuppression duration and accumulation of cancerous cells. Parameters are presented in Table 1. *c_n_* and *d_n_* are modified along axes

First, we observe that several short immune challenges could lead to larger accumulation of nonhealthy cells than a long episode lasting for the same total duration of immunosuppression (quantified by the product between number of immunosuppressive challenges and their duration) (Fig. 3). The positive relation between total immunosuppression and number of cancerous cells accumulated (Fig. 3 Inplot) suggests that the role of acute challenges is worthy of consideration.

**Figure 4.**
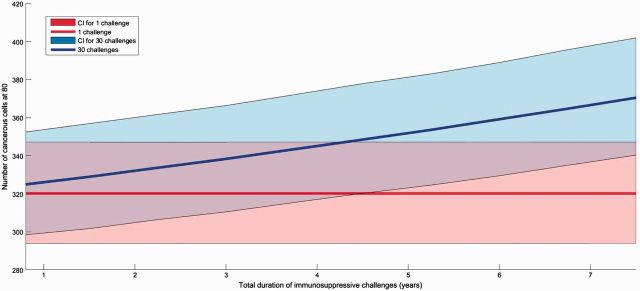
Influence of the number of immunosuppressive infections on the accumulation of cancerous cells. For a total immunosuppression duration indicated on *x* axis (in years), red area shows that a single immunosuppressive infection has almost no influence of number of cancerous cells at the end of individual life. On the opposite, blue area shows the sharp increase in this abundance of cancerous cells when immunosuppression is distributed over 30 short infections. The maximal accumulation of cancerous cells for 30 challenges and a total duration of 7.5 years is of 370 cells. Parameters are detailed in Table 1. Areas are confidence intervals quantified by a Latin Hypercube Sampling (LHS) with 100 iterations allowing testing sensitivity for a 5% change in all parameter values and solid lines represent the median value obtained from LHS

Then, to confirm this observation, we explored two different scenarios where challenges can be persistent or acute and evenly repeated 30 times. We found that a single long immunosuppression challenge leads to a very small change in cancerous cells accumulation while 30 repeated short challenges covering the same total duration are expected to produce a sharper increase in the proliferation of cancerous cells (Fig. 4). These conclusions are robust to sensitivity analysis and also hold when partial immunosuppression of weaker amplitude is considered (Supplementary Fig. S1).

### Influence of immune activation challenges combined with immunosenescence

**Figure 5.**
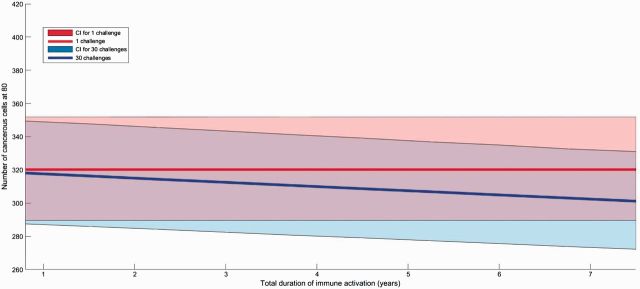
Influence of the number of immune activation following infections on the accumulation of cancerous cells. For a total immune activation duration indicated on *x* axis (in years), red area shows that a single infection has almost no influence of number of cancerous cells at the end of individual life. Blue area shows the slight decrease in this abundance of cancerous cells when immune activation is distributed over 30 short infections. The minimal accumulation of cancerous cells for 30 challenges and a total duration of 7.5 years is of 305 cells. Parameters are detailed in Table 1. Areas are confidence intervals quantified by a Latin Hypercube Sampling (LHS) with 100 iterations allowing testing sensitivity for a 5% change in all parameter values and solid lines represent the median value obtained from LHS

Noticing the significant effect of repeated immunosuppressive challenges on accumulation of cancerous cells, we then looked at the influence of immune activation challenges on the same estimation of cancer risk. With our realistic parameters, we found that a long period of immune activation slightly reduce the number of cancerous cells (Supplementary Fig. S2). In addition, 30 repeated immune stimulations lead to a higher decrease of cancerous cells than a single long one for the same total duration (Fig. 5 and Supplementary Fig. S3), but with weak amplitude. Since we assume that immune system efficiency cannot be higher than before immunosenescence, the increase of immune system activation cannot be of the same magnitude than negative effects (e.g. an increase of 70% could result in a ‘net’ increase much lower because the maximal activity is constrained by the immune system efficiency before immunosenescence beginning, as show in Fig. 1).

## DISCUSSION

Our model describes the paradoxical role of immune challenges on cancer risk with a particular emphasis on the neglected role of acute challenges (i.e. alteration of immune efficiency for <6 months). These immune challenges can be beneficial for cancerous cell proliferation when they downregulate the adaptive immune response to cancer (called immunosuppressive challenges) or detrimental to cancerous cell proliferation when they upregulate this immune pathway (immune activation challenges). First, our model predicts that repeated acute immunosuppressive challenges may increase cancer proliferation in a greater extent than a persistent one for the same total immunosuppression duration. Frequent immunosuppressive episodes, combined with immunosenescence, may result in the immune systems’ failure in controlling cancer cell’ growth and density, due to immunosuppressive episodes occuring prior to the recovery of maximal elimination of cancerous cells. In contrast, repeated short immune activation episodes could slightly reduce the accumulation of cancerous cells compared to a single persistent challenge. Regular activation of the immune system could offset the action of immunosenescence and therefore may offer a protection regarding age-related cancerous cell accumulation.

As for any modeling approach, our model is based on a series of simplifying assumptions that deserved to be discussed. First of all, as dynamics and cross-talk with the immune system could be different for congenital and acquired cancer, further studies need to assess the influence of immune challenges on cancerous cell accumulation for each of them. Then, we assumed that the immune system removes cancerous and precancerous cells in an identical manner, whatever their phenotype. This should be relaxed in future studies regarding the huge diversity of cancerous and precancerous cells [41], which suggests that immune effectors can specifically target only some cancerous clones. Third, we made the hypothesis that immunosenescence follows a purely gradual process, while it is possible that nonlinear relationships exist between age and immune function, especially in the very elderly [42]. To take into account this issue, we tested different immunosenescence curve shapes but they do not significantly change our results (Supplementary Fig. S4). The parameter values chosen in this study may influence the quantitative outcomes of our theoretical framework, but we would like to point out that our conclusions are robust to changes in the parameter space (as shown in Supplementary Data).

While more realistic and complex models can be compared with empirical data, we nevertheless believe that our simple and general model can nevertheless provide a number of testable predictions on how immune challenges may affect the risk of malignancy via the immune system. Indeed, a lot of uncertainties are documented on what could be the impact of each component of the immune system on cancerous cell proliferation [13]. Therefore, a model with a greater complexity will have to deal with a lot of speculation about each of these components, decreasing its relevance to study transient immune challenges over cancer progression. While this should be a natural next step of our research work, it was then important highlighting this possibility through a simple model.

In our model, the risk of cancer is approximated by the number of cancerous cells at 80 considering that it is the end of life. Indeed, more abnormal cells individuals have, more the risk to develop cancer symptoms during life will be high. In the current state of our knowledge, our estimator seems the most parsimonious but few others could be used as: probability to having a cell with a certain number of aberrations or probability to have one metastatic cell. In order to have a global view of the influence of immune challenges on carcinogenesis, further studies should investigate and compare our results with these different estimators of cancer risk.

We found a maximal accumulation of ∼10^3^ cancerous cells (all scenarios confounded) which correspond to a tumor of 0.01 cm according to the growth tumor curves in [43] or even larger [44]. Nevertheless, such size would be under detection threshold. Because our goal here was to address only the impact of multiple immune challenges, we did not consider any additional factors that could strongly amplify cancerous cell accumulation (exposure to toxics, pollutants and genetic predisposition) up to a detectable tumor size. For multi-factor diseases like cancer, it was important to highlight the impact of multiple challenges alone before considering how their effects could be combined with other susceptibility or proliferation factors. Studying such combination of different processes represents an intuitive extension of this study.

As infections are an important source of immune challenges, their frequency and duration should be correlated with the diversity of pathogenic agents. Thus, they may represent a key tool to explore links between cancer and immune challenges. In fact, the long-term impact of persistent immunosuppressive infections on cancer risk has already been supported by several studies based on clinical data. Especially, it is widely recognized that adults infected by Human Immunodeficiency Virus (which can persist ∼60 years even with treatment [45]), have an increased risk of malignancies as lung cancer [46]. In addition, several other persistent viruses (i.e. Epstein Barr virus and Cytomegalovirus) could result in a persistent immunosupression by exploiting/destroying immune cells (B cells and macrophages, respectively) or by active secretion of immunomodulatory molecules [47, 48].

The originality of our study is to predict that acute immunosuppressive infections could also impact cancer risk and in a larger extent than persistent infections. Empirical evidences of such situation are obviously harder to identify, but the impact of ‘common’ diseases on immune system and their relation with cancer risk are worthy of investigation. In fact, a protein secreted by influenza A virus (pandemic flu) inhibits IFNβ expression and therefore suppresses both innate and acquired immune responses [49]. In addition, other common viruses as rhinovirus, responsive of common cold and rotavirus, agent of gastroenteritis, have also been associated with a immune deficiency in infected people [50, 51]. As individuals may experience several episodes of flu, common cold and gastroenteritis during their course of life, these numerous short induced immunosuppression periods will probably not be neutral concerning the accumulation of cancerous cells. However, history of common colds or gastroenteritis prior to cancer diagnostic has been associated with a decreased cancer risk in a cohort study [52]. It may suggest that infections have a complex impact on immune responses to cancer and that further studies need to consider the temporal dynamic of immune challenges following the entry of an infectious organism. Finally, a complete and persistent immunosuppression following infections seems unlikely and latent infections (EBV and Herpesvirus) could rather produce short immunosuppression challenges each time they reactivate.

Conversely, our results suggest that multiple immune activations across life could decrease cancer risk comparing to a single one. The discontinuity theory proposed by Pradeu and colleagues [53] could give an explanation to this result. The theory states that immune responses are induced by the appearance of molecular motifs that are different from those with which the immune system has regularly interacted so far and could be tolerant regarding to motifs that are persistent. As a matter of fact, apparent protection against lung cancer has been observed in humans frequently exposed to cattle in the dairy industry [54]. It is possible that this protection is provided by endotoxins present in the dust which are known to be potent immune stimulating factors [55]. Moreover, evidences of acute infections being antagonistic to cancer has been reviewed by [56].

We do believe that this study could be the first step to envision innovative guidelines for cancer prevention and identification of groups at risk for cancer. Impacts of immune challenges are particularly worth of interest to study the observed disparity of cancer incidences between low and high income countries. Our results suggest a stronger impact of acute and repeated immune challenges after the beginning of immunosencence. This situation could be applied to high-income countries where longer lifespan have been shown to induce chronic low-grade inflammation, contributing to immune disorders in older individuals [57]. Even if poor-quality of available data and the comparatively shorter life expectancy may explain lower cancer incidence in low-income countries [58], we suggest that it could also be link to the frequency and the nature of immune challenges (numerous short periods of immune activation). It may also depend on variability of individuals’ immune system (see Supplementary Fig. S5). In fact, it has been shown that variation in the human immune system is largely driven by nonheritable influences [59]. Depending on their environment, individuals will: (i) have different quantity of energy available to invest in their immune responses and (ii) meet different infectious burden and thus different levels of selective pressure to develop a fully efficient immune system [60]. In addition, antigenic exposure early in life through common infections is recognized to be essential for establishing an immunological memory [61]. All these sources of variation may impact the frequency and the time of infection but they could also directly impact the probability to develop cancer.

Finally, exploring the consequences of frequent immune challenges could become an interesting alternative way to design more integrative public health strategies, moreover regarding the issue of chemotherapy resistance that puzzles the scientific community since decades and the development of immunotherapy strategies. 

## Supplementary data

Supplementary data are available at *EMPH* online.

## Supplementary Material

Supplementary Data
