# Supplementary material for: Interactions between immune challenges and cancer cells proliferation: timing does matter!
Source: Evol Med Public Health. 2016 Aug 18;2016(1):299–311. doi: 10.1093/emph/eow025 (PMC5046994; doi:10.1093/emph/eow025)
Supplement: Supplementary Data [file eow025_Supp.doc]

**Supplementary materials**

**Impact of weaker immunosuppression amplitude**

In the main text, we show that repeated short immunosuppressive infections have a larger impact on cancerous cells than a single long one. Nevertheless, we assumed in the main text that immune system activity is decreased by 70% during infections. Figure S1 shows that an infection decreasing immune system activity by only 10% of its capacity leads to similar conclusion, even if the pattern is less striking.


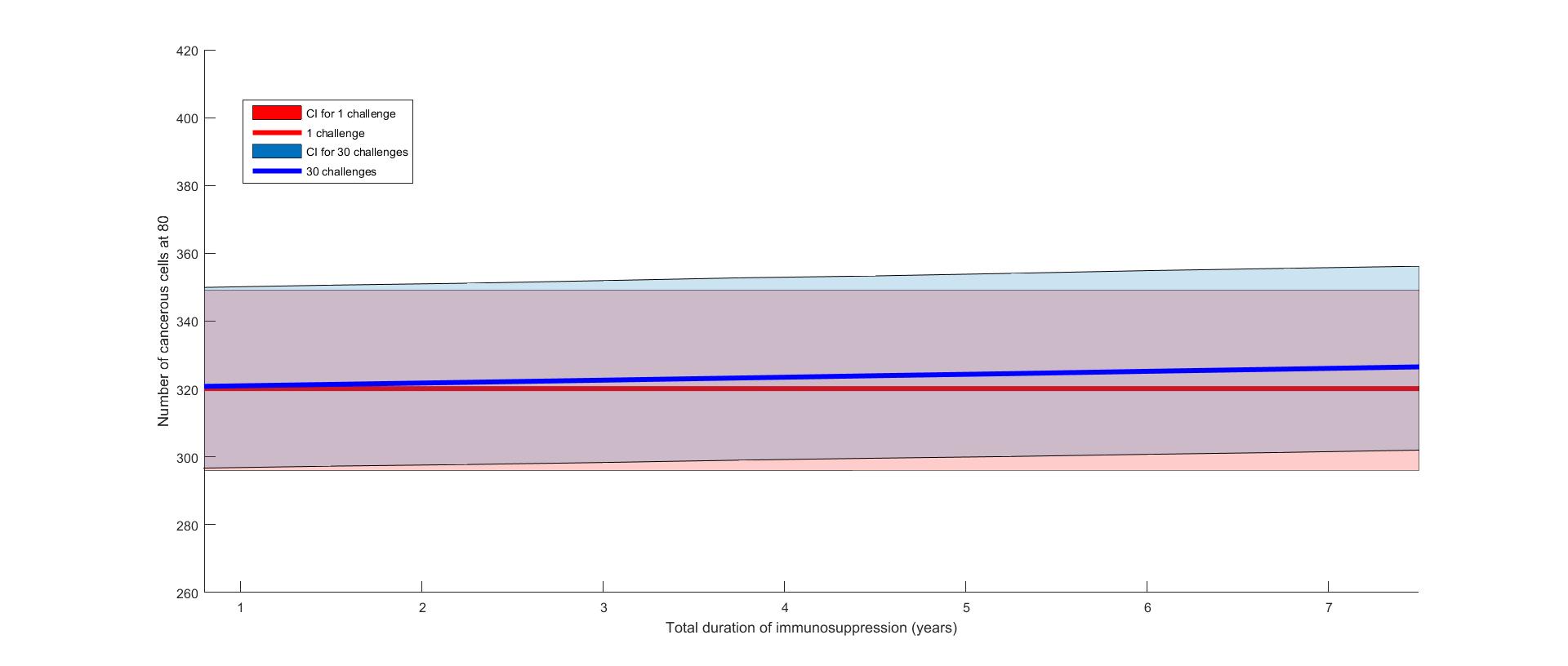


Figure S1: Same figure than Figure 4, but with an amplitude of immunosuppression of 10% instead of 70% (a_3_=0.1).

**Influence of immune activation following infections on the accumulation of cancerous cells.**

**
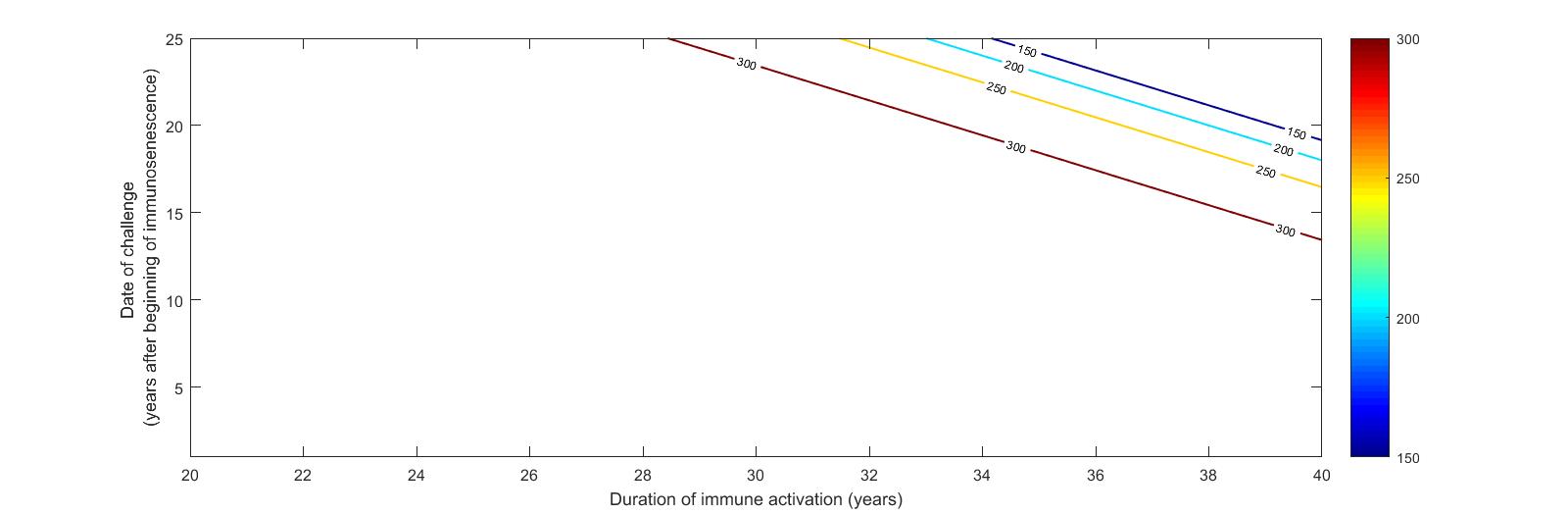
**

Figure S2: Same figure than Figure 2, considering an immune activation following the infection (a_3_=-0.7).

**
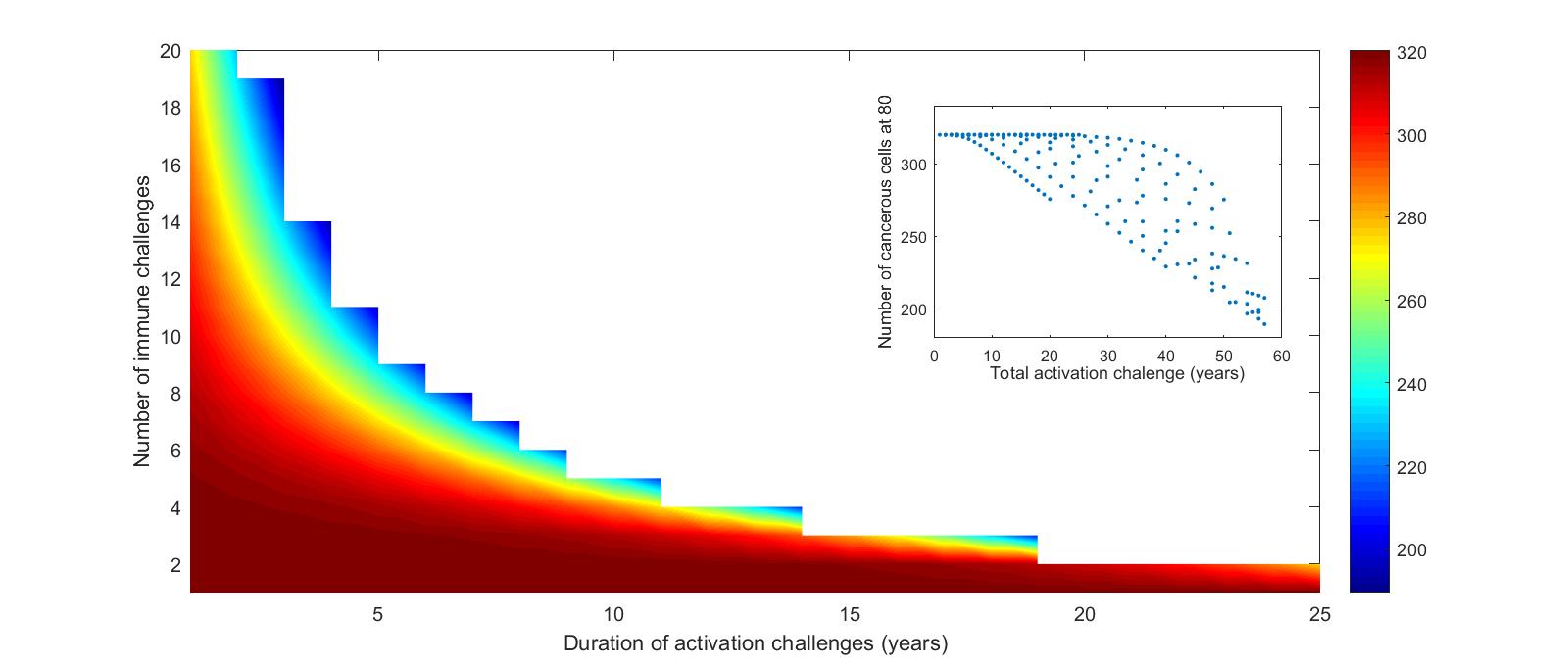
**

Figure S3: Same figure than Figure 3, considering an immune activation following the immune challenge (a_3_=-0.7).

**Impact of later immunosenescence start**

For our principal results, immunosenescence has been started at 20 according to the beginning of thymic output reduction (Table 1). However telomere length of CD4+ T cells decline around 45 years [1]. Figure S4 shows that a later immunosenescence stat to similar conclusions for immunosuppressive infection and the greater accumulation of cancerous cells in the case of 30 repeated acute infections is even more striking.

**
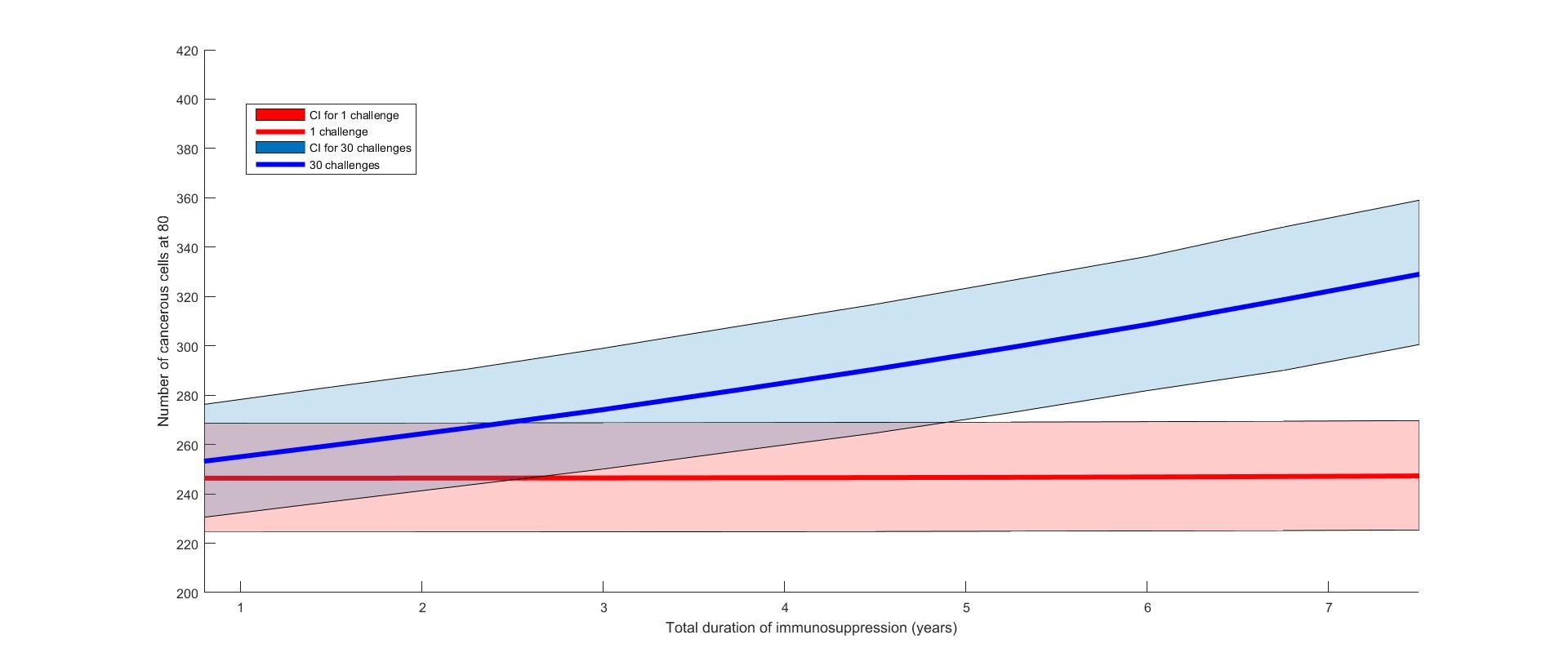
**

Figure S4: Same figure than Figure 4, but here immunosenescence starts at 45 instead of 20 (b_0_=45).

**Impact of initial strength of immune system**

For our principal results, we assumed that immune system efficiency before the beginning of immunosenescence allows the elimination of 70% of cancerous cells (*a_1_,* Table 1). However, the immune system of individual is extremely variable depending on available energy and personal history of infection. Figure S5 shows that a weaker basal immune efficiency leads to similar conclusions for immunosuppressive infection.


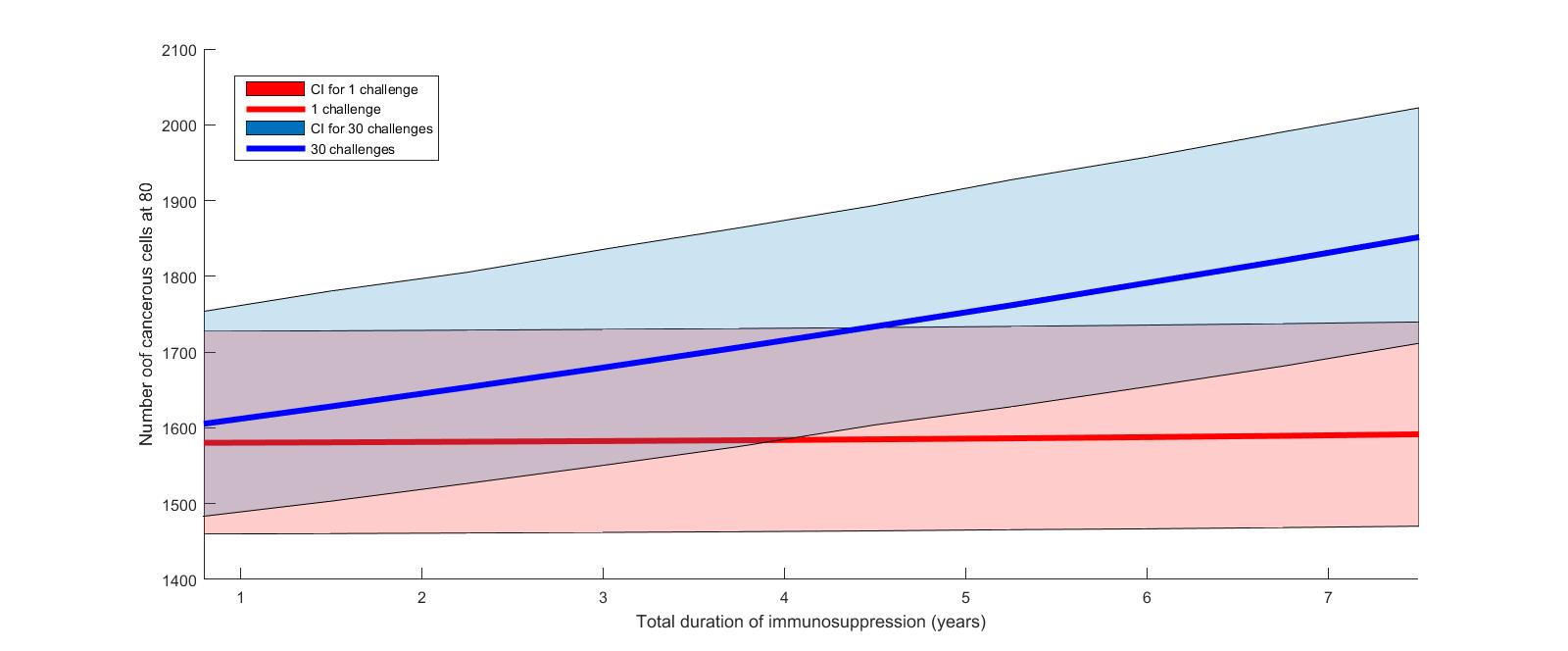


Figure S5: Same figure than Figure 4, but here immune efficiency before immunosenescence is of 40% (a_1_=0.4).

1. Aydar, Y., Balogh, P., Tew, J. G. & Szakal, A. K. Age-related depression of FDC accessory functions and CD21 ligand-mediated repair of co-stimulation. *Eur. J. Immunol.* 2002 ; **32**, 2817–26.
